# Bronchoscope Size Selection for Improved Diagnostic Yield in Peripheral Pulmonary Lesions: A Retrospective Study

**DOI:** 10.7759/cureus.79260

**Published:** 2025-02-18

**Authors:** Kohei Okafuji, Atsushi Kitamura, Yutaka Tomishima

**Affiliations:** 1 Department of Pulmonary Medicine, Thoracic Center, St. Luke's International Hospital, Tokyo, JPN

**Keywords:** a diagnostic yield, bronchoscope size selection, bronchoscopy, ebus-gs, peripheral pulmonary lesions

## Abstract

Introduction

There is uncertainty about choosing a bronchoscope size for approaching peripheral pulmonary lesions (PPLs). This study aimed to compare the circumstances of using thick and thin scopes and determine the optimal approach for better diagnostic yields.

Methods

We retrospectively reviewed patients who underwent bronchoscopy for PPLs with thick or thin bronchoscopes at St. Luke’s International Hospital between April 2011 and December 2014. We compared the clinical characteristics of patients with thick and thin bronchoscopes.

Results

A total of 220 patients underwent bronchoscopy for PPLs; 121 patients (median age 69 years, range 20-94 years) were included. The thick bronchoscope group (n = 67) and thin bronchoscope group (n = 54) were similar in age, sex, and PPL shape and location, but not in size. Diagnostic yield was significantly higher in the thick group (79.1% vs. 59.3%, p = 0.0271). There were no significant differences in the diagnostic yield for upper-lobe PPLs (74.4% vs. 67.6%, p = 0.615), but the thick group had significantly higher diagnostic yields for lower-lobe PPLs (84.6% vs. 43.8%, p = 0.014). When the endobronchial ultrasonography findings were adjacent to or invisible, there were significant differences (75.0% vs. 46.2%, p = 0.0498).

Conclusion

For PPLs located in the lower lobe or if a probe cannot display a within-position, thick bronchoscopes should be preferentially chosen.

## Introduction

In recent years, various bronchoscopic techniques, such as endobronchial ultrasonography using a guide sheath (EBUS-GS) and virtual bronchoscopic navigation (VBN), have been developed and reported to improve the diagnostic yield for peripheral pulmonary lesions (PPLs) [1-11]. Additionally, it has been reported that the addition of transbronchial needle aspiration through a guide sheath with endobronchial ultrasonography (GS-TBNA) is effective in diagnosing PPLs, particularly when lesions are not identifiable on radial endobronchial ultrasonography (R-EBUS) [12-14]. Although these advancements have improved diagnostic yields, there remains a lack of clear guidelines regarding the selection of bronchoscope sizes, despite their varied availability. Thick bronchoscopes offer the advantage of acquiring larger specimens and are preferable for selecting molecularly targeted drugs, which have gained importance in recent years [15]. However, they may lack selectivity in navigating the bronchi. Conversely, thin bronchoscopes provide greater precision in bronchial selection, facilitating easier access to lesions, with reports highlighting the utility of ultrathin bronchoscopes [16,17].

Despite the distinct advantages and disadvantages associated with each bronchoscope size, uncertainty remains regarding the circumstances favoring the use of thick or thin bronchoscopes in approaching PPLs. Consequently, bronchoscopists often rely on personal experience and preferences when selecting an appropriate size. Knowing in advance which bronchoscope size to choose under what circumstances could potentially enhance the diagnostic yield and streamline examination procedures. Therefore, this study aimed to delineate the optimal approach for selecting bronchoscope size to improve the diagnostic yield in cases of PPLs.

## Materials and methods

Study design and patients

This single-center, retrospective, observational study was based on a review of the medical records of patients who underwent bronchoscopy to diagnose PPLs at St. Luke's International Hospital between April 2011 and December 2014. Patients who underwent bronchoscopy with an intermediate bronchoscope, such as BF260 (Olympus, Tokyo, Japan), or an ultrathin bronchoscope, such as Xp260 (Olympus), were excluded from the study.

This study was conducted in accordance with the principles of the Declaration of Helsinki. St. Luke’s International University Clinical Research Ethics Committee approved the study protocol on June 10, 2024 (approval number: 24-R036). Because the data were anonymous, the requirement for informed consent was waived.

Thick and thin bronchoscopes

We categorized the bronchoscopes into two groups: thick bronchoscopes, designated as 1T240, 1T260, and 1TQ290 (Olympus, Tokyo, Japan) (Group L), and thin bronchoscopes, represented by P260 and P290 (Olympus) (Group S). Table 1 displays the specific features of each bronchoscope.

**Table 1:** Characteristics of thick and thin bronchoscopes Group L, thick bronchoscopes; Group S, thin bronchoscopes

Bronchoscope	Tip diameter, mm	Bending angle, ° (Up / Down)
Group L		
1TQ290 (n = 26)	5.9	180 / 130
1T260 (n = 33)	5.9	180 / 130
1T240 (n = 8)	6.0	180 / 130
Group S		
P290 (n = 12)	4.2	210 / 130
P260 (n = 42)	4.0	180 / 130

Procedures

Prior to the examination, we utilized a 1.25 mm thin slice computed tomography (CT) and VBN with LungPoint® (Broncus Medical Inc., Mountain View, CA, USA) to estimate the bronchial path leading to the lesion. Based on the CT data acquired at a thickness of 1.25 mm, we created a virtual bronchoscopic route indicating the path to the target lesion.

All bronchoscopies were performed under local anesthesia. Pretreatment involved pharyngeal anesthesia with 5 mL of 2% lidocaine, followed by moderate sedation with an initial dose of pethidine (25 mg) and midazolam (1.0-3.0 mg), with the dose of midazolam determined based on the patient’s age, body size, and comorbidities. The bronchoscope was orally inserted, with intratracheal 2% lidocaine. To maintain moderate sedation during the procedure, additional doses of pethidine (25 mg per dose) and midazolam (0.5 mg per dose) were administered as necessary.

Lesions were identified using R-EBUS performed with an endoscopic ultrasound system (EU-ME1, Olympus) equipped with a 20 MHz mechanical radial-type probe of 1.4 mm (UM-S20-17S, Olympus) or 1.7 mm (UM-S20-20R, Olympus) diameter. For the thick bronchoscope, a 1.7 mm probe and guide sheath (external diameter, 2.55 mm; SG-201C, Olympus) were used, while a 1.4 mm probe and guide sheath (external diameter, 1.95 mm; SG-200C, Olympus) were used for the thin bronchoscope. An endobronchial ultrasound (EBUS) probe with a guide sheath was inserted into the lesion through the bronchoscope’s working channel and advanced toward the target lesion under the fluoroscopic guidance of the C-arm. If the lesion could not be identified using R-EBUS, a curette (CC-6-DR-1; Olympus) was used to guide the sheath. Once the lesion was identified using R-EBUS, the probe was withdrawn, and the guide sheath was positioned in the lesion for specimen collection.

Four cytology specimens were initially collected using a brush (BC-204D-2010, BC-202D-2010; Olympus), with one specimen used for rapid cytological examination with Papanicolaou staining to confirm successful lesion retrieval. Subsequently, biopsies were performed using forceps (FB-233D: maximum outer diameter; 1.5 mm, FB-231D: maximum outer diameter; 1.9 mm, Olympus) for histological examination. Biopsies were repeated until an adequate number and size of specimens were obtained, with at least five tissue specimens collected per case. If the lesion could not be identified using R-EBUS with a thick bronchoscope, GS-TBNA with a 21G needle (NA-1C-1; Olympus) was added. This procedure was performed as described by Kitamura et al. [18].

Bronchoscope selection

**Figure 1:**
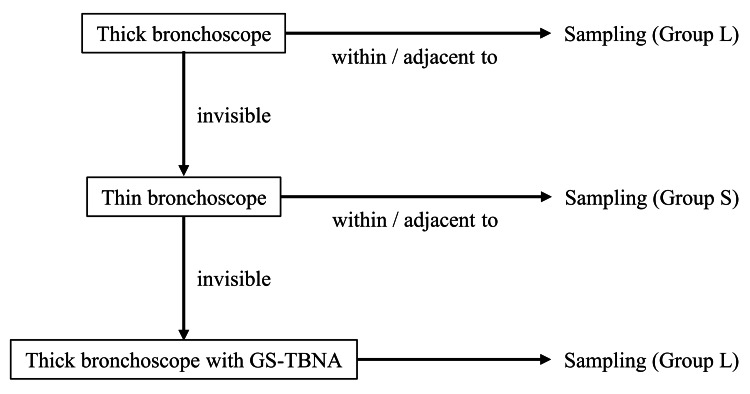
Bronchoscope selection and group classification based on EBUS findings Initially, a thick bronchoscope was used for specimen retrieval if the lesion was identified on R-EBUS. If R-EBUS failed to detect a lesion, a thin bronchoscope was used to improve bronchial selection. In cases where R-EBUS with a thin bronchoscope identified a lesion, the specimen was obtained directly. If R-EBUS with a thin bronchoscope failed to detect the lesion, a thick bronchoscope was reintroduced for GS-TBNA. Patients were classified based on the size of the bronchoscope used at the time of final specimen collection. Group L, thick bronchoscope; Group S, thin bronchoscope Abbreviations: EBUS, endobronchial ultrasound

Initially, a thick bronchoscope was used to obtain a large specimen. A thick bronchoscope was used for specimen retrieval if the lesion could be identified on R-EBUS. However, if R-EBUS failed to detect a lesion, the bronchoscope was switched to a thin one to facilitate improved bronchial selection. If R-EBUS identified a lesion using a thin bronchoscope, the specimen was directly obtained. In cases where R-EBUS with a thin bronchoscope could not identify the lesion, we reverted to a thick bronchoscope for specimen collection, aiming to perform GS-TBNA. The efficacy of GS-TBNA in the diagnosis of PPLs has been reported, particularly in cases in which lesions remain elusive to R-EBUS [12-14]. Patients were categorized based on the size of the bronchoscope used at the time of final specimen collection. In cases where multiple bronchoscopes were used during the procedure, the group classification was determined by the bronchoscope used for the final successful sampling (Figure 1).

Diagnosis

The final diagnosis was based on pathology, microbiology, and the clinical course. Cases that could not be diagnosed with bronchoscopy were determined radiologically and clinically, either through alternative diagnostic procedures or after a minimum of 2 years of post-bronchoscopy follow-up.

Statistical analysis

We utilized an unpaired Student’s t-test to compare the means of continuous variables and Fisher’s exact test to compare the proportions of categorical variables between the groups. P-values < 0.05 were considered statistically significant. All statistical analyses were performed using the EZR version 1.68 software (Saitama Medical Center, Jichi Medical University, Saitama, Japan) [19].

## Results

Patients and PPLs

Out of the 220 patients who underwent bronchoscopy for PPLs between April 2011 and December 2014, those who met the exclusion criteria were excluded. The exclusion criteria were as follows: 95 patients who underwent bronchoscopy with an intermediate bronchoscope, two patients who underwent bronchoscopy with an ultrathin bronchoscope, and two patients for whom the size of the bronchoscope used could not be determined. After applying these exclusion criteria, 121 patients remained eligible for the study. Among them, 67 were categorized into Group L and 54 into Group S. Patient demographics were comparable in terms of age, sex, and lesion characteristics such as shape and location. However, the size of the PPLs was significantly larger in Group L. Furthermore, Group L exhibited significantly better EBUS findings (Table 2).

**Table 2:** Characteristics of patients and peripheral pulmonary lesions Unpaired Student's t-test was used to compare the means of continuous variables, and Fisher's exact test was employed to analyze the proportions of categorical variables between the groups. Group L, thick bronchoscope; Group S, thin bronchoscope Abbreviations: GGN, ground glass nodule; EBUS, endobronchial ultrasound

Variables	Group L (n = 67)	Group S (n = 54)	P-value
Age, years (median, range)	69 (20-94)	67 (39-89)	0.86
Male, n	45	28	0.096
Lesion size, mm (median, range)	25 (9-125)	16 (6-44)	0.0000028
Lesion shape, n			0.053
Solid nodule	47	42	
Pure GGN	1	3	
Part-solid nodule	10	9	
Consolidation	4	0	
Cavity	5	0	
Lesion location, n			0.12
Right upper lobe	15	24	
Right middle lobe	2	1	
Right lower lobe	17	12	
Left upper lobe	24	13	
Left lower lobe	9	4	
EBUS findings, n			0.0251
Within	36	16	
Adjacent to	24	16	
Invisible	4	10	

Diagnostic yields

In Group L, 53 and seven patients were diagnosed using bronchoscopy and surgery, respectively. Conversely, in Group S, bronchoscopy was used to diagnose 32 cases, and surgery was used to diagnose 14 cases. Group L had a significantly higher diagnostic yield than Group S (79.1% vs. 59.3%). The final diagnoses of each group are presented in Table 3.

**Table 3:** Final diagnoses Group L, thick bronchoscopes; Group S, thin bronchoscopes

Diagnoses	Group L, n	Group S, n
Lung cancer		
Adenocarcinoma	29	26
Squamous cell carcinoma	8	4
Small cell carcinoma	6	2
Not otherwise specified	5	6
Atypical carcinoid	1	0
Metastatic lung cancer		
Breast cancer	0	1
Colon cancer	1	0
Sclerosing hemangioma	1	0
Nontuberculous mycobacteria	1	1
Mycobacterium tuberculosis	2	2
Aspergillosis	1	0
Lung abscess	1	0
Inflammation	6	4
Unknown	5	8

When comparing diagnostic yields based on the lesion site, excluding the middle lobe, due to the small number of cases, there was no significant difference between Group L and Group S with 74.4% and 67.6% for the upper lobe. However, for the lower lobe, the yield was 84.6% for Group L and 43.8% for Group S, indicating a significantly higher yield for Group L (Table 4).

**Table 4:** Diagnostic yields Unpaired Student's t-test was used to analyze the differences between the groups. Group L, thick bronchoscope; Group S, thin bronchoscope Abbreviations: EBUS, endobronchial ultrasound

Variables	Group L	Group S	P-value
Overall, %	79.1 (53/67)	59.3 (32/54)	0.0271
Lesion location			
Upper lobe, %	74.4 (29/39)	67.6 (25/37)	0.62
Lower lobe, %	84.6 (22/26)	43.8 (7/16)	0.014
EBUS findings			
Within, %	83.3 (30/36)	81.3 (13/16)	1.00
Adjacent to / Invisible, %	75.0 (21/28)	46.2 (12/26)	0.0498

Regarding the diagnostic yields based on EBUS findings, there was no significant difference between Groups L and S (83.3% vs. 81.3%) when the lesions were identifiable. However, when lesions could not be identified, the diagnostic yield was significantly higher in Group L than in Group S (75.0% vs. 46.2%) (Table 4).

## Discussion

In our study, the overall diagnostic yield was higher in Group L than in Group S, particularly for lesions located in the lower lobe and those that could not be identified with R-EBUS, whereas the yields were similar for lesions in the upper lobe. The higher diagnostic yield observed in Group L could be attributed to the initial use of a thick bronchoscope to obtain large tissue specimens. It is plausible that there were more accessible cases in Group L, as the identification of lesions with a thick bronchoscope allowed for specimen retrieval without necessitating the use of a thin bronchoscope. Indeed, lesions in Group L were significantly larger and more cases in Group L were identified using R-EBUS (Table 2).

Group L exhibited a significantly higher diagnostic yield than Group S in the lower lobe, whereas the yields were comparable for the upper lobe. This disparity in the lower lobe can be explained by our initial use of a thick bronchoscope and the relatively straight anatomy of the lower-lobe bronchus, which facilitated bronchial selection. However, the thick bronchoscope still achieved a sufficiently high diagnostic yield of 84.6% (Table 4), and from the perspective of obtaining larger tissue samples, the strategy of prioritizing the use of a thick bronchoscope for lower-lobe lesions appears to be reasonable. Conversely, in the upper lobe, despite our strategy of using a thick bronchoscope, the diagnostic yields were similar between groups L and S. This may be because the upper-lobe bronchus has steeper angles, making it more difficult to select bronchi. Moreover, the P290 bronchoscope offers a 210° bending angle, enhancing flexibility (Table 1), which might have been advantageous for bronchial selection in the upper lobe.

In cases where lesions could not be identified with R-EBUS, the diagnostic yield was significantly higher in Group L, suggesting that GS-TBNA contributed to the improved yield. Notably, four cases were diagnosed only with GS-TBNA and not with other specimen collection methods. Based on our findings, we propose that initiating bronchoscopy with a thick bronchoscope for lower-lobe lesions and a thin bronchoscope for upper-lobe lesions could enhance the diagnostic yield and streamline examination, potentially reducing the need for bronchoscope changes mid-procedure.

A previous study compared the diagnostic yields of thick and intermediate bronchoscopes, showing that intermediate bronchoscopes were more effective [20]. Similarly, a study comparing thin and ultrathin bronchoscopes demonstrated the superiority of ultrathin bronchoscopes [21]. However, no prior research has compared the diagnostic yields of thick and thin bronchoscopes, as done in this study, which highlights the significance of these findings.

Previous studies have consistently reported the usefulness of thin bronchoscopes [16,17,22,23], while there have been no studies specifically evaluating the effectiveness of thick bronchoscopes. This focus on thin bronchoscopes is understandable given their ability to navigate more peripheral airways. However, these studies have not addressed the importance of obtaining larger tissue samples for selecting molecularly targeted therapies in cancer [24], and it has also been reported that the analysis of next-generation sequencing for specimens biopsied using small forceps is prone to be unsuccessful due to an insufficient amount of nucleic acid [15]. If the diagnostic yield of thick bronchoscopes is comparable, they may be preferable for this purpose. Additionally, this study introduces the novel concept of using thick bronchoscopes for lesions in the lower lobes, representing a valuable new insight.

However, this study has several limitations. First, it was a retrospective, non-randomized controlled trial. Second, because our study was conducted at a single institution, our findings may not be generalizable. Third, multiple bronchoscopists performed the study, which led to variations in skill levels. Fourth, this study was not a direct comparison between cases in which a thin bronchoscope was used from the beginning and those in which a thick bronchoscope was used from the beginning. Therefore, caution is required when interpreting the statistical results.

## Conclusions

This study is the first attempt to delineate a strategy for selecting an appropriate bronchoscope size. Our findings suggest that employing a thick bronchoscope as the initial approach, particularly when lesions cannot be identified with R-EBUS or when they reside in the lower lobe, and opting for a thin bronchoscope primarily for upper lobe lesions, can enhance the diagnostic yield of PPLs. These insights could serve as practical guidelines for bronchoscopy selection in clinical practice.
